# Ways of integrating eating into everyday lives – a qualitative study in Germany

**DOI:** 10.1186/s40795-024-00883-5

**Published:** 2024-05-21

**Authors:** Lyn Lampmann, Agnes Emberger-Klein, Katrin Brückner, Klaus Menrad

**Affiliations:** TUM Campus Straubing for Biotechnology and Sustainability, Chair of Marketing and Management of Biogenic Resources, Weihenstephan-Triesdorf University of Applied Sciences, Straubing, Germany

**Keywords:** Food-related behaviour, Eating action, Qualitative type-building content analysis, Typology, Personality development

## Abstract

**Background:**

Food-related behaviour is a very complex topic. A common way to reduce complex issues to their essential content is to create a typology. In Germany, with regard to food-related behaviour, the creation of a typology has often been carried out by commercial research institutes, but also by (international) scientific institutes. The former have mostly used quantitative methods, the latter usually have a specific content focus. Within this study, we want to investigate how people integrate eating into their everyday lives while engaging with themselves and the environment, thereby living out personality development and related socialisation.

**Methods:**

37 qualitative interviews were conducted and evaluated by means of content-structuring qualitative analysis and type-forming qualitative content analysis. Interviewees were recruited via recruitment calls using different channels, such as newspapers or university e-mail lists. Participants over the age of seventeen were eligible to take part in the study. Both the individual action processes and the interpretation processes regarding food-related behaviour were taken into account. The final sample consisted of 20 male and 18 female participants with an age range from 18 to 83.

**Results:**

The result were seven eating action types, namely: Eating as a way of life, The Relaxed, Eating as self-determination, Eating as a necessary Evil, The Adaptive, The Overstrained and The Controlled.

**Conclusions:**

We contribute to the study of food-related behaviour with the chosen qualitative method of type-building and by looking at how people integrate eating into their everyday lives. This contributes to a broader understanding of this phenomenon and complements the findings of existing commercial and academic food-related typing-building activities. Specifically, through identifying “The Overstrained” as a novel eating action type, this study adds to the literature in the field and may be a useful baseline for future research.

**Supplementary Information:**

The online version contains supplementary material available at 10.1186/s40795-024-00883-5.

## Background

‘Food has not only biological, but also social, cultural and psychological functions. Food is important for social relationships and communication, as it can signal friendship, belonging and closeness. It can also indicate social status, power, hierarchy and marginalisation and is an expression of religious, ethical and moral beliefs. Food and eating can generate self-esteem and emotional security, but also fear and feelings of guilt.’ ( [1] – own translation). All these realms of everyday life concerning individual as well as social levels can be influenced by food-related behaviour, thus making this a very complex issue [2–4]. In previous research, various disciplines have attempted to reduce the inherent complexity of food-related behaviours.

To streamline investigations like food-related behaviour, constructing a typology is an effective strategy [5–7]. Typologies categorise subjects or objects based on shared characteristics, simplifying data complexity into manageable and meaningful categories [8, 9]. This method is widely used in both quantitative research, such as cluster analysis, and qualitative research to lessen the intricacy of diverse behaviours [5]. In the realm of commercial consumer research, especially for understanding food demand, creating nutritional typologies is a standard practice.

In Germany, commercial market research institutes [10–13] have developed different nutrition types to explain the development of food demands, such as, The Nestlé Study “Climate and nutrition” 2021 [14]. The segmentation study developed six climate nutrition types being *uninvolved sceptic, uncompromising activist, concerned preserver, active individualist, prudent observer, reserved orientation seeker*. Frequently, these nutrition types developed by market research institutes and/or by big companies are rather different among each other and the applied methods for their development often are not disclosed. Nonetheless, commonly multivariate statistical methods are applied examining the consumers regarding their differences or similarities [15].

In addition, nutritional types are built in several (international) scientific studies [16–21]. In [22, 23], these types of nutrition are explained in more detail. As an example, the study conducted by Jelenko et al. [24] is demonstrated here. The study involved 60 guided interviews with people from urban areas as well as guided interviews and group discussions with 20 people from rural areas. The approach was to find requirements for sustainable nutritional practices. The following types were analysed using grounded theory: *altruism and family, resource orientation and efficiency, ecology and social criticism, tradition and home, individualism and distinction, desire and emotion, body and illness, health promotion and mental strength*.

Jelenko et al. [24] as well as the other academic typologies concentrate on following special thematic focuses: sustainability [21], pleasure [19], healthy eating [16], sensory preferences [20], obesity [18], or the requirements for sustainable nutritional practices in everyday life [24]. Most of the studies apply quantitative survey tools [16–18, 20, 21] or combine qualitative and quantitative measures [21]. In contrast to the commercial and academic nutritional types mentioned above, we look at how food is integrated into everyday life to draw conclusions about people’s personalities. Thus, the analysis does not concentrate on circumstances but rather on the personality traits of individuals themselves that are reflected in food-related behaviour. Looking back at the opening quote, this is crucial due to the emphasis that food has components of both inner and outer reality [1]. Our understanding of the human being is based on the socialisation theory of Hurrelmann and Bauer [25], which is described in the “Introduction to Socialisation Theory” (“Einführung in die Sozialisationstheorie” [25]). There, the authors describe the development of personality as a definitional component of socialisation, describing the ‘lifelong acquisition of and confrontation with the natural endowments, especially the basic physical and psychological characteristics, which represent the “inner reality” for humans, and the social and physical environment, which represent the “outer reality” for humans’ ( [25] – own translation). These realities are not created and invented, but people have a methodical and epistemological access to inner and outer reality. The confrontation with inner and outer reality plays a crucial role in the development of personality [25, 26].

The qualitative research paradigm advocates a humanistic view of human beings, in which people have free will and the need for self-realisation [27, 28]. That is why we let a person talk freely without aiming for a specific question or answer when it comes to capturing an unfiltered and authentic approach to food-related behaviour and the way people actively and consciously interact with themselves and their environment. Findings from this study enable us to understand the differences and similarities [29] of how people develop their personality at least partly through their food-related behaviour and how their personality is expressed through their own food-related behaviour. In order to adequately address the complexity of the topic, types are build using type-building qualitative content analysis [30]. This complements the findings of existing commercial and academic nutritional types, on the one hand, for example in terms of what circumstances are required to avoid obesity, and, on the other hand, what has to be considered regarding the personality of the person. In a further study [31] we then look at the implicit and explicit motives (by Julius Kuhl [32]) of our participants in order to find answers on why people developed these certain types of personality. With our studies, we operate in the field of social psychology. We assume that individual food-related behaviour serves to shape personality, but above all to express personality. We want to find out what this looks like in detail. The research question is therefore: how do people integrate eating into their everyday lives while engaging with themselves and the environment, thereby living out personality development and related socialisation?

## Methods

### Data collection

In 2017, the first author conducted 42 qualitative semi-structured, problem-centred interviews. They were carried out at the Chair of Marketing and Management of Renewable Resources at the City of Straubing, at the Institute for Biomedicine of Aging in Nuremberg, or at the home of the interviewees either in the City of Straubing or in the City of Regensburg. The participants were contacted by means of a university e-mail distribution list, a recruitment call in the local newspapers or a list of participants from previous studies.

The guideline for the interviews was developed after reviewing relevant literature about eating types. It consisted of six sections: the introduction including the informed consent, the application of the Operant Multi Motive Test (OMT) [31], a section about food-related behaviour including a 24-Hour-Recall and a problem-centred interview, the application of the Personality Research Form (PRF) and a short questionnaire about socio-demographic characteristics and a conclusion. The present manuscript concentrates on the data about food-related behaviour. We pretested the interview guideline twice and adjusted it slightly. All interviews were tape-recorded, except one, in which notes were taken [33, 34].

All participants gave informed written consent and their participation was voluntary. The Data Protection Officer of the HSWT approved the data privacy statement and the documentation of the procedure. Additionally, we followed the ethical principles of the Helsinki Declaration, the German Research Foundation (DFG) and the German Society for Sociology (DGS). At the beginning of the interviews, we emphasised that there were no right or wrong answers to avoid social desirability regarding a seemingly optimal nutrition. It has also been pointed out that the interviewer was no nutritionist with detailed knowledge in the nutrition sector, but a social scientist and that the subjective structures of meanings regarding eating were of main interest.

The section dealing with food-related behaviour started with the 24-Hour-Recall [35] about the previous day’s diet as a stimulus to facilitate the introduction to the problem-centred interview and to activate the interviewees. We used pictures that showed a variety of typical groceries in order to support the memory, to visualise the diet day and to avoid palliations. We also offered inscribable proxy cards for the groceries that were not represented on our pictures. Due to the assumption that a normal weekday might be nutritionally different to a weekend-day, participants were additionally asked to describe the diet of the previous Sunday. If the previous day was a Sunday, participants had to describe the previous Friday as the weekday [21]. Examples of the 24-Hour-Recall can be found in the appendices (Figs. 1 and 2).

In order to create a methodological structure of the problem-centred interview, these were structured by the two dimensions of the *eating action approach*. For Spiekermann [36] food-related behaviour should be understood as *eating action* (“Esshandeln” [36]), since he describes this term as a combination of interpretation and action processes of active and self-thinking people. It considers experiences of individuals and their self-contained rationality [36]. It complies with our understanding of the human being by Hurrelmann and Bauer [25] and covers the entire sphere of the experience of food intake, including social references and ambience, thus, all perceptions during and after the meal [37]. We understand the interpretation processes as covering the inner reality and the action processes as covering the outer reality. Therefore, the Theory by Hurrelman and Bauer [25] was referred to as a methodological framework to cover their understanding of personality development. The action processes look at what people *actually do* regarding their food-related behaviour (action processes; “Handlungsprozesse” [36]), and the interpretation processes at how people *interpret* their action processes (interpretation processes; “Deutungsprozesse”; [36]). This *eating action approach* is then also the foundation for the feature space, which is described in the section *Data coding and analysis.*

To cover the action processes questions about the type of groceries that were eaten, the cooking behaviour and arrangement of the eating situations and the snacking behaviour were asked. Questions about the significance that eating captures in everyday life, the emotions connected with eating, the meaning of eating in company and inner conflicts regarding eating covered the interpretation processes. Enquiries were always possible in the sense of a semi-structured interview [38] if more detailed information was needed. In the case of enquiries, the first author worked with the technique of paraphrasing, that is, the interviewer summarised the spoken word to reflect whether she had understood it correctly. Finally, all interviewees answered a questionnaire about socio-demographic characteristics [33, 34]. The section about food-related behaviour took between 21 min and 61 min, with an average duration of 38 min.

All data has been anonymised to ensure that participants are not identifiable through their given information.

### Sampling and participant information

In the recruitment process, care has been taken to form a relatively heterogeneous group of interviewees regarding age and to divide males and females fairly. The only inclusion criterion for the interviews was an age higher than 17 years, due to privacy aspects and the need for parental consent for younger participants.

37 of the interviews could be considered for the study. The other five were not considered due to incomplete or unusable questionnaires, a different nationality that we decided afterwards not to consider in terms of comparability, and due to the mentioning of an eating disorder that we did not interpret as belonging to normal food-related behaviour. However, this number of interviews proved to be sufficient, as no new themes could be found during the interviews, which was confirmed by the data analysis. Thus, saturation was reached [39]. None of the selected persons refused to take part, nor withdrew during the interviews. All interviewees received €15 as appreciation for their participation. The youngest person was 18 and the oldest two were 83 years old. In total, we had 20 men and 18 women with a Body Mass Index (BMI) range between 19.0 and 34.4. Accordingly, 27 participants had normal weight, eight participants were pre-obese, and three participants were obese according to the BMI [40]. All participants were Germans. Thus, they were socialised with Western European culture. Three of the participants (all female) were vegan, three were vegetarians (one male, two female) and 31 were omnivore.

### Data coding and analysis

The interviews were audiotaped and transcribed verbatim following the rules of Dresing and Pehl [41]. The first author was the key person for the coding and analysis. Initially, qualitative content analysis in the form of a content structuring qualitative analysis was applied to analyse the data, as described by Kuckartz [30] using MAXQDA as coding software [42]. This form of analysis aims to identify topics and sub-topics and their systematisation and conceptualisation.

We worked with the deductive-inductive approach, whereby following the guideline, existing (deductive) as well as new (inductive) thematic (close to the material) and analytical (conceptualising, abstracting) categories were generated and identified [30]. The result was a hierarchical code system [30] with 15 main categories and several sub codes each. Once the categories had been created, we went through all interviews and assigned suitable text passages to the categories. To ensure that the assignment undertaken was not subjective, the consensual coding technique was applied, so that two persons coded the interviews independently and compared their results afterwards [30]. Finally, the elicited categories were assigned either to the action processes or to the interpretation processes.

Next, a typology of *eating action* was built by following Kuckartz’ [30] type-building content analysis. This procedure describes a methodological controlled analysis, in which a typology is the result of a grouping process where a certain social condition is divided into types by means of one or more features. As a result, the elements of one type are as similar as possible (internal homogeneity), but as different as possible compared to other types (external heterogeneity) [30, 43].

Then we wrote case summaries for each interview considering Spiekermanns *eating action approach* [36] and the two dimensions of “action processes” and “interpretation processes”, which form the feature space (“Merkmalsraum”; [44]) for the typology. In doing so, we worked fact-oriented and close to the text from the perspective of the research question [30].

To develop the typology, the case summaries were distributed fairly to five independent researchers, who again had to summarise the case studies in key words according to the two dimensions of the *eating action approach*. During two team meetings, the group members presented one after another their cases, i.e. subjects, regarding the important features and pinned them on a pin board – either next to or away from the already presented and similar or varying cases. The group constantly discussed the results. Through this procedure, certain groups evolved [30]. The result was a coarse structure of the typology. The next step was to free the emerged types from their assigned cases. Then the first, second and fourth authors differentiated the emerged types and afterwards assigned the cases again to the types [45] to construct polythetic types, which means the cases that belong to one type are as similar as possible but must not be identical [30]. The result are real types [45]. All developed types are construct of model cases that are the synopsis of the best-suited text segments. For this purpose, relevant text segments are selected from different cases according to the criterion of plausibility for the type to be described and presented together to describe the one specific type [30]. The characteristic differences between the eating action types, analysed using a mixed-methods approach, are described in detail elsewhere [22]. Furthermore, implicit motives of each eating action type are discussed in another study [31].

## Results

The analysis of the problem-centred interviews and the typing resulted in seven *eating action types.*

In the following, all *eating action types* are described in detail and their main and subthemes are summarised in Table 1. Furthermore, essential quotes to underline the findings as well as essential aspects of their food-related behaviour are summarised.

**Table 1:** Main and subthemes of the eating action types

Eating Action Type	Main theme	Subthemes
*Eating as a way of life* (*n* = 3)	Idea of ideal nutrition, self-imposed rules for food-related behaviour	Concepts, such as veganism or diets mainly based on (wild) herbs; full endorsement into the concept with food-related behaviour having a high priority; high emphasis on eating according to their own personal and bodily needs; the acquisition of information makes it possible to develop their own food concept; the individual idea and the associated behaviour, and the consumption of corresponding food leads to a good conscience
*The Relaxed* (*n* = 7)	Conscious and relaxed, effortless implementation of food-related concepts	Food-related principles are performed; food is strongly connected to positive emotions which sometimes leads to eating more than physically needed; development of their own health concepts, whereby feeling comfortable with the ideas of what they consider to be an appropriate food-related behaviour regardless of actual effects
*Eating as self-determination* (*n* = 7)	Food is given high priority since it is the means to satisfy the self-related needs	The self is the focal point of this type and their own needs and claims get special attention; development of self-made nutritional concepts with special focus on healthy nutrition or a healthy saturation; exceptions to the actual consumption behaviour are considered fine as long as they serve the satisfaction of own needs
*Eating as a necessary Evil* (*n* = 3)	Little involvement in food-related behaviour	Eating and everything that belongs to it has low importance; little thought is given to food, eating and food-related behaviour; emotionless about eating; rare cooking frequency due to the lack of motivation to cook only for oneself; preference for simple meals
*The Adaptive* (*n* = 5)	Adaptation to others	Inactive until other people become active or decide how to act; cooking is especially fun when other people are involved; development of own health concept which can be flexible and adapted according to the (social) situations
*The Overstrained* (*n* = 8)	Overstraining due to either internal overstraining with eating or external circumstances like unemployment or illness	Internal debates about correct behaviour; feelings of insecurity about food-related behaviour; weight control is important: there is a strong mental confrontation with the fear of weight gain or with the discomfort of the current physical condition; lack the feeling for a subjectively adequate and good diet resulting in negative assessment of own food-related behaviour
*The Controlled* (*n* = 4)	Compulsion that forces this type to keep control of their body	Parts of the body control refer to weight control, a strongly represented aspect in this type; balance behaviour by doing sports or self-imposing restrictions; believe in natural bodily concerns, which are expressed through statements such as listening to the own body

### Eating as a way of life

Generally, individuals of *Eating as a way of life* (3 assigned persons: 2 females, 1 male) give food-related behaviour a high priority.

Importantly, the handling of one’s own claims towards the social environment is emphasised:

“*My inner conflict in this context, of course, is how you then perceive other people in relation to their eating habits. If I accept what the other person eats and thus accept him/her in the same way. Do I then also accept the habits he/she has and say with it that it is not actually bad; because that would be a contradiction with my own attitude. And I found out for myself someday that I can just accept a person for his/her humanity, but still find habits he/she has as not good. And then I actually get along quite well with that. You can also like other people and spend time with them and still say this or that behaviour that you show there is not good and that was the breakthrough to be able to sit at the table with other people while they eat their steak or something*.” (Concept: veganism).

It is very important not to be constrained or influenced by external factors. Possible needs are for example the need for a sensible handling and intake of food. This aspect explains why people of this type often grow their own fruit and vegetables in their gardens. The essential requirement of a sensible intake of food is connected to expediency and salubriousness but also gratitude, as pointed out:

*Well, I am very grateful for the food I have and that I am able to buy organic food. Because I know a lot of people who would like to do that, but they don’t have the money. (…) Yes, because I perceive life more consciously now. I don’t know/ I just think I’d say to eat more consciously and to be grateful for it*.

### The Relaxed

A conscious and relaxed, relationship towards their food-related behaviour describes *The Relaxed* (7 assigned persons: 4 females, 3 males).

They also perceive cooking for only oneself as too costly. Nonetheless, this type formulated the demand for a warm (and self-made) meal once a day with food from their own garden. Thereof, people of this type prepare food in batches to meet their own ideas. These ideas are summarised in the following statement:

“*But as a rule, I plan the weekend in such a way that I have the weekend in advance to really have something warm on the table for lunch, something I cooked myself. Yes. And I just try to put a lot of emphasis on good food, of course on what we grow ourselves*.”

If these ideas are fulfilled and the food is taken in sensibly, people emphasise the resulting good conscience:

“*I feel good and comfortable with it, firstly because I know that I enjoyed it, secondly because I had time, that I could enjoy it, that I wasn’t under any time pressure and thirdly because I believe that I also fed myself well and correctly and varied*.”

### Eating as self-determination

For people of *Eating as Self-determination* (7 assigned persons: 5 females, 2 males), the focus is on the self, implicitly asking: What is doing me good right now?

Highlighted is the claim for self-made food, as follows:

*“Basically, I feel better when the food comes from home or when I made it myself or my mum made it*.”

Homemade food receives special attention from this type. The resulting associations are consistently positive. This circumstance shows once again the importance of self-determination for this type: because only those who cook for themselves really have control over what they eat.

### Eating as a necessary evil

The type *Eating as a necessary Evil* (3 assigned persons: 2 females, 1 male; average age is 81.6 years) often eats together with other persons because of the importance attached to social interactions and the fact that food is more likely to be neglected. However, this type reports on weight control, although this does not really influence or stress the individuals in their daily lives, as the following quote underlines:

*“Don’t have any problems. Only that I still have to bring the weight down*.”

Individuals of this type do not connect any positive emotions with food and eating, as follows:

*“So [it = eating] is necessary for the preservation of life, but there is no fun in it. I can’t say.”*

The fact that food does not play a major role in everyday life of this type is not perceived as disturbing or regrettable. If the latter were the case, people of this type would not behave as they do.

### The Adaptive

The individuals belonging to this type simply have to participate. The adaption to other people by *The Adaptive* (5 assigned persons: 1 female, 4 males) becomes clear by the following statement:

*“Okay, and that’s where I adapt. Then I eat white bread or baguette or on weekends […] the only son who still lives in […] comes with his wife and one of my grandchildren […] and they bring, I pay, but they bring the food. Therefore, they determine […].”*

The citation also shows that there are hardly any individual demands, which are consistently implemented in their own food-related behaviour.

Eating together with other (close) people gets special attention, as the following quote emphasises:

“*So I think that […] the dining room is such a common place or the place of meeting and where one does something together, takes meals together, sits at a table and in peace and not shoo, shoo, between door and hinge/ no, I don’t like that. And yes, [there] is not a side table somewhere in the kitchen […], but in the dining room […] at the dining table and the table is set properly and […] yes, and eaten together. And it doesn’t matter whether it’s the children and me or all four of us.”*

### The Overstrained

Generally, *The Overstrained* (8 assigned persons: 2 females, 6 males) is often stressed when it comes to nutritional issues, as for example by saying:

*“And sometimes it’s stress because when I can’t decide what I want, it stresses me. Especially before shopping, because I always think about what I have to buy or what I want to buy and that’s a mixture of I’m actually happy that I can buy everything I want, because I don’t have anything at home and on the other hand it’s like: buy the RIGHT one too*.”

Nevertheless, this type also expresses its joy or anticipation of eating:

*“It’s like, for example, if we go somewhere to eat where I know it’s delicious and I know the menu, then I’m very happy and happy all day long and I think about what I’m going to eat tonight or what I feel like eating.”*

The obvious contradiction between stress and anticipation towards the food-related behaviour is a clear indication of the overstraining. The quote underlines the importance attached to food and eating, which is the reason for the overstraining: only something that means a lot to someone can make that person unhappy if he or she has not yet managed to develop its appropriate implementation.

### The Controlled

*The Controlled’s* (4 assigned persons: 2 females, 2 males) main motive is to control their body, as mentioned in the following quote:

*“But even if I’m hungry, I still don’t eat. Because I always pay attention to my kilos. But I actually like doing it (eating) very much*.”

The statement clearly shows that control behaviour is perceived as self-imposed limitation. In addition, a characteristic behaviour for this type is to balance previous behaviour by actions such as sports or food restrictions, as the following quote shows:

*“Or [doner kebab], I always think, only the content would be enough already. But well, I always weigh it up, what have you eaten today, what have you done, HAVE you done something, have you only sat at your desk or not, so you go for a walk*.”

Both sports and dietary restrictions as compensatory behaviour are accompanied by intense mental debates. Some of these debates relate to cognitive reactions caused by eating. Here, the focus is on weighing up processes, e.g. with regard to the question whether dinner should be taken late in the evening at all.

## Discussion

In this study we have concentrated on how personality is (at least partly) developed and expressed through food-related behaviour. In order to give the inner and outer reality that brings up personality a methodological structure that is based on nutritional theory, we considered how people actually eat (action processes – outer reality) and how they interpret (interpretation processes – inner reality) their own food-related behaviour. Our qualitative approach resulted in seven qualitative *eating action types*, named *Eating as a way of life*, *The Relaxed, Eating as self-determination, Eating as a necessary Evil, The Adaptive, The Overstrained* and *The Controlled*. In the following, we discuss the types considering current insights into food-related behaviour and insights about personality development.

*Eating as a way of life* reflects personalities, who want to move freely within their food-related ideas. This indicates the need for self-realisation [46], which can be understood as an important personality trait of this type. Klotter [46] assumes that ‘People are well aware of the artificiality of lifestyles, i.e. they are self-aware and can choose or reject them.’ [3]. In the expression of personality in the context of food-related behaviour, the body and food are spiritualised, both are woven into a web of meaning. People of this type become ecological or ethical and feel like their minds are winning over their bodies [46]. Hence, the emphasis of the type’s own demands (e.g. sensible food intake) belongs to differentiation mechanisms [45], since personality development is also a matter of external perception, as it develops through the interaction of individuals with the environment [25, 46]. By interacting with others, one’s own food-related behaviour is strengthened, while external influences on it are categorically rejected.

The food-related behaviour of *The Relaxed* can be interpreted as the ability to eat in moderation. Dijker [47] explains the most important components for moderate food-related behaviour, namely perception (mindful interaction with other people and food), awareness (the belief in the special nature of food) and motivation (cooking skills). Moreover, this type seems to have a presumably benevolent view of things. The presence of these components is expressed in moderate food-related behaviour without self-control and without sacrificing the pleasure of eating. Pleasure, joy and anticipation are associations that are repeatedly mentioned by *The Relaxed*. In addition, Dijker [47] emphasises one’s own craft as a reason to understand the motivation to cook. Although people of the relaxed type sometimes lack the motivation to cook, they have found a way to realise craftsmanship by cooking in batches.

By *Eating as self-determination*, food-related behaviour is understood as a possibility for self-determination and the associated well-being [48]. Compared to *Eating as a way of life*, this type is not concerned with self-realisation but with self-determination as an important personality trait. This type focuses on optimizing situations for their well-being, which also leads to making food-related exceptions. In the self-determination theory (SDT), motivation is the energy to act [48]. *Eating as self-determination* works in the sense of autonomous motivation [48], as people of this type show strong self-motivation to satisfy their needs using available resources or alternatives, respectively [49]. This enables them to feel good and also explains spontaneous consumer behaviour, as it promotes creativity, problem solving, achievement and positive emotions [48].

*Eating as a necessary Evil* differs from the other types as eating and everything that belongs to it has no special relevance for this type. It is reasonable to assume that the non-existent desire to eat is age-related. Scientific literature links loss of appetite and malnourishment to old age [50–52], yet people of the “Eating as a Necessary Evil” type experience neither. They lack the importance that food and eating has for most people. People of this type put special effort into spending time with important people like family and friends, since ‘Ageing (…) poses an increased risk of isolation and lack of social interaction, particularly at meal times.’ [53]. This is problematic insofar as the decline in relationships in old age is caused by factors that are beyond individual control (structural losses in the circle of friends and acquaintances, legal regulations on retirement) [54]. Therefore, emotional states such as loneliness and loss may appear. In short, people of this type actively try to keep alive the remaining social relationships they have and rate the value of eating as secondary. The personality trait here appears to be social interactions as an essential aspect of well-being. One could assume that personality is lived almost exclusively through outer reality.

What is striking is that this is similar to *The Adaptive*: this type needs social interactions (for example eating situations) to be able to give their personality expression. For social adaption, three essential aspects are required: identity preservation in the course of adaption, the perception of the adaption as desired action and the existence of appropriate circumstances enabling the subject to implement the required changes [55]. Thus, if people adapt to others, it must ‘allow individuals to maintain their socio-cultural nature, their personality, and life purposes.’ [55]. They must therefore feel comfortable with the adaptation, reflected by the three aspects mentioned above. Thus, for *The Adaptive*, food-related behaviour can be understood as one of many areas, where living in accordance with their own personality by adapting to others comes into play.

Both, *The Adaptive* and the *Eating as a necessary Evil* are somewhat guided by their social environment. While *Eating as a necessary Evil* craves social meaning for their food-related behaviour, *The Adaptive* often conforms to their social surroundings. It is therefore reasonable to assume that there is a type that is *The Adaptive* in social situations, but *Eating as a necessary Evil* when they are on their own, since adaption is part of the personality and cannot be lived out when being alone. Therefore, eating situations may not have any importance when being alone. This connection would also widen the age range of the *Eating as a necessary Evil* type.

An overstraining with the subjectively correct implementation of food-related behaviour marks *The Overstrained*, leading to perceiving their action possibilities as somehow limited. Literature gives diverse reasons for the feeling of limitations ending in overstraining [56–58], such as a discrepancy between implicit and explicit motives. Indeed, *The Overstrained* repeatedly emphasised the discrepancy between inner values, ideas and desires and external circumstances and demands. Job et al. [59] showed that motivational discrepancy is related to emotional distress, which may be responsible (at least in part) for the link between motivational discrepancy and food-related behaviour. People with motivational discrepancy eat more and prefer unhealthy, tasty foods because they want to downregulate the emotional distress caused by motivational discrepancy.

Maintaining control characterises *The Controlled* in terms of personality. Sociological theories of action assume the body to be a controllable instrument that is subservient to the will of human beings [60, 61]. According to Sieber [62], the winning types of our time swim, run and fight, for ‘muscular, well-trained, fitness strong’ [60] bodies represent positive connoted social norms such as e.g. achievement, perseverance or strength and shape the thinking of people, strengthen social conventions and represent effects of social power [60, 63]. Foucault [63], for instance, describes the body to be the object and target of power. Sports and a balanced diet are the main practices to achieve an athletic body [64]. Thus, for *The Controlled* it can be assumed that apparent power is gained through the satisfaction of social norms by self-control. This kind of conviction and behaviour can be recognised as inherent to this type.

A major and important result of this study is the identification of *The Overstrained* as an independent *eating action type*. This behavioural pattern cannot be confirmed by other studies, where overstraining occurs within other types, but does not describe an independent food-related behaviour due to the personality trait of being overstrained by the discrepancy between implicit and explicit motives [10–13, 17, 19, 21]. As Hayn et al. [65] and Jastran et al. [66] explain, everyday activities must be implemented actively; they must therefore be constructed, stabilized, maintained and changed by the individual. In this study, it was suggested that *The Overstrained* failed to actively implement everyday food-related behaviour due to perceived internal or external constraints, which prevent the development of a stable personality.

In summary, it is possible to understand the differences and similarities [29] of how people develop their personality at least partly through their eating behaviour and how their personality is expressed through their own eating behaviour. What is special about the seven *eating action types* is the focus on the status quo of the personality traits of the respective people, which are expressed through their food-related behaviour. In a further study, we looked at how the development of the different personalities can be explained [22]. As seen in the results, different ways of how to develop personality and socialise successfully with the everyday action of how to integrate eating into everyday lives exist. Only *The Overstrained* builds an exception by not managing to integrate a feel-good kind of food-related behaviour. The reason may be a disturbed personality development and a failed socialisation due to a conflict between internal or external constraints. However, it is equally possible to overcome the feeling of being overstrained. The feeling should therefore not be understood as static, but as dynamic, which can be changed with appropriate support. This is an important new finding of our study, that could not only inform future research, but also dietitians. Moreover, with regard to *Eating as a way of life*, it would be interesting for nutritionists to develop eating situations in which these people would feel self-realising, if the need exists to change food-related behavioural patterns. For *Eating as self-determination*, it would be important to offer a scope of action or alternatives from which people of this type can choose in order to continue to feel self-determined and at the same time influence their food-related behaviour if necessary. For people of the type *Eating as a necessary Evil* it is highly important to create feel-good social situations if there is a need to change food-related behaviour or if wished to offer new products (maybe especially invented for the needs of elderly). The same applies to *The Adaptives*. At the same time, measures need to be developed to enable people of this type to make appropriate eating decisions on their own, such as supporting eating alone. With regard to *The Controlled*, it is important to recognise the meaning of power in order to understand why and how food-related behaviours are formed in everyday life and how these can possibly be influenced. Future research could build on the findings of this study by developing practical tools, such as surveys, that could be used by dietitians and researchers to assess the *eating action types* of individuals.

Finally, in the sense of limitations, we cannot make any claim to general external validity with 37 interviews conducted, which would also contradict the qualitative research approach. Additionally, we can relate our results only to Germany and they might differ in other countries. In addition, we have committed ourselves to the goal of building polythetic types. Nevertheless, there are individual differences depending on the people assigned within the types. In each case, we had to decide how to deal with this aspect. The results show the outcome of this decision-making process.

## Conclusions

The seven *eating action types* of this study are characterised by an individual-centred approach that leaves the voice with the subject and allows for a detailed description of people’s eating action. Eating is an everyday act, which makes it complex, but it also influences many areas of everyday life and can be used to develop and express personality.

With our qualitative type-building approach, we could reduce the complexity of and structure how people integrate eating into their everyday lives, while engaging with themselves and the environment. Our study therefore provides useful findings for nutritional advice, for marketing purposes and for socio-cultural research, which can use the results to develop practical life and nutritional solutions for food-related (mis)behaviour. By taking into account the findings of other studies that focus on external circumstances in combination with our findings that take into account people’s personality traits, meaningful situations and also products can be created that can have a positive influence on people’s food-related behaviour.

However, in order to be able to compare the results of this study with other cultural circles, further research is necessary, using quantitative methods, if necessary, in order to be able to make a claim to comparability.

### Electronic supplementary material

Below is the link to the electronic supplementary material.

Supplementary Material 1

## Data Availability

The datasets used and analysed during the current study are available from the corresponding author on reasonable request.

## Abbreviations

OMT: Operant Multi Motive Test
PRF: Personality Research Form
HSWT: Hochschule Weihenstephan-Triesdorf
BMI: Body Mass Index
SDT: Self Determination Theory
