# Supplementary material for: Ways of integrating eating into everyday lives – a qualitative study in Germany
Source: BMC Nutr. 2024 May 21;10:76. doi: 10.1186/s40795-024-00883-5 (PMC11106892; doi:10.1186/s40795-024-00883-5)
Supplement: Supplementary file 1 — Supplementary Material 1 [file 40795_2024_883_MOESM1_ESM.docx]

Appendices


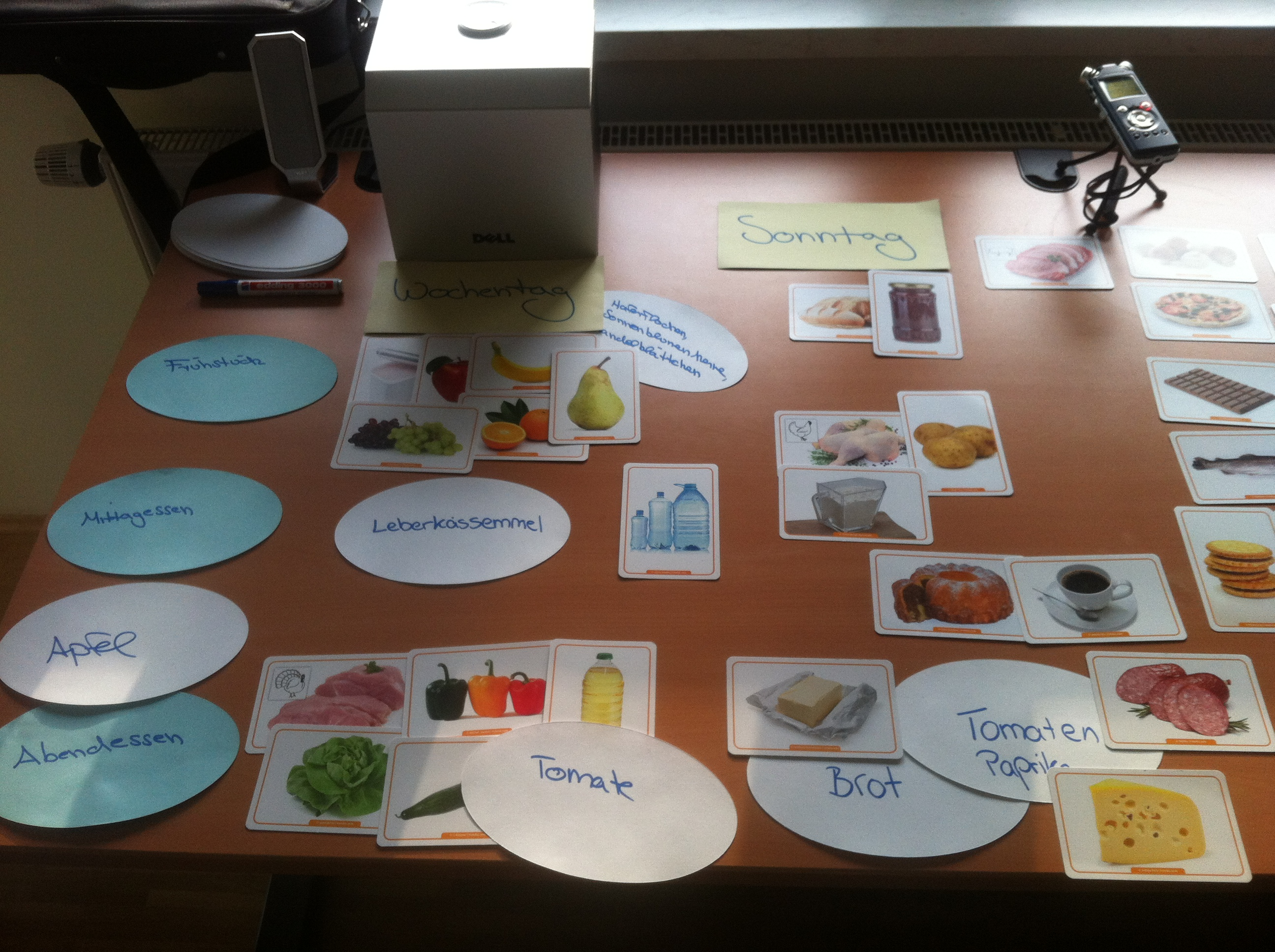


Figure 1. Example of the 24-hour consumption recall using photographs and written notes.


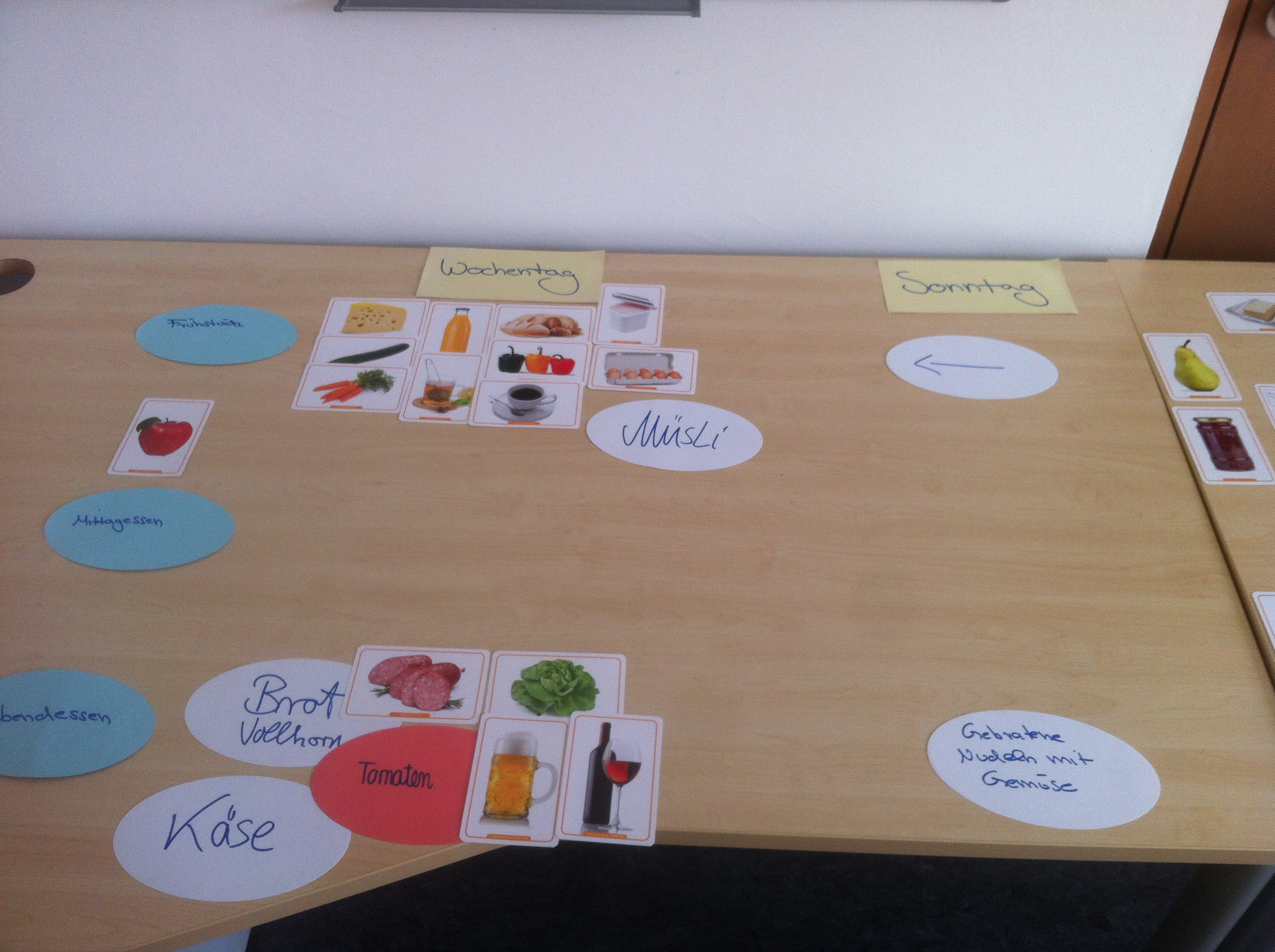


Figure 2. Example of the 24-hour consumption recall using photographs and handwritten notes.
